# The survival outcomes and prognosis of stage IV non-small-cell lung cancer treated with thoracic three-dimensional radiotherapy combined with chemotherapy

**DOI:** 10.1186/s13014-014-0290-7

**Published:** 2014-12-18

**Authors:** ShengFa Su, YinXiang Hu, WeiWei Ouyang, Zhu Ma, Bing Lu, QingSong Li, HuiQin Li, ZhiYong Wang, Yu Wang

**Affiliations:** Department of Thoracic Oncology, Affiliated Hospital of Guiyang Medical College; and Guizhou Cancer Hospital, Guizhou, People’s Republic of China; Department of Thoracic Oncology, Gui zhou Cancer Hospital, 1 Beijing Road West, Guiyang, 550004 People’s Republic of China

**Keywords:** Non-small cell lung cancer, Stage IV, Three-dimensional radiotherapy, Prognosis

## Abstract

**Background:**

The impact of thoracic three-dimensional radiotherapy on the prognosis for stage IV non-small-cell lung cancer is unclear. This study is to investigate survival outcomes and prognosis in patients with stage IV non-small cell lung cancer (NSCLC) treated with thoracic three-dimensional radiotherapy and systemic chemotherapy.

**Methods:**

Ninety three patients with stage IV NSCLC had received at least four cycles of chemotherapy and thoracic three-dimensional radiotherapy of ≥40 Gy on primary tumors. The data from these patients were retrospectively analyzed.

**Results:**

Of the 93 patients, the median survival time (MST) was 14.0 months, and the 1, 2, and 3-year survival rates were 54.8%, 20.4%, and 12.9%, respectively. The MST of patients received radiation dose to primary tumor ≥63Gy and <63 Gy for primary tumor were 15.0 and 8.0 months, respectively (P = 0.001). Patients had metastasis to a single site and lower tumor volume (<170 cm^3^) also produced longer overall survival time (*P* = 0.002, *P* = 0.020, respectively). For patients with metastasis at a single site, thoracic radiation dose ≥63 Gy remained a prognostic factor for better overall survival (P = 0.030); patients with metastases at multiple sites, radiation dose ≥63 Gy had a trend to improve overall survival (P = 0.062). A multivariate analysis showed that radiation dose ≥63 Gy (P = 0.017) and metastasis to a single site (P = 0.038) are associated with better overall survival, and the volume of primary tumor was marginally correlated with OS (P = 0.054).

**Conclusions:**

In combination with systemic chemotherapy, radiation dose ≥63 Gy on primary tumor and metastasis to a single site are significant factors for better OS, aggressive thoracic radiotherapy may have an important role in improving OS.

## Background

Approximately 55% of patients who have been newly diagnosed with non-small-cell lung cancer (NSCLC) have distant metastases [1]. For NSCLC patients with stage IV disease and good performance status, platinum-based combination therapy improves survival and quality of life, and four to six cycles of chemotherapy are recommended [2]. Thoracic radiotherapy is often used as a palliative treatment for patients with stage IV NSCLC to relieve symptoms (i.e. hemoptysis, cough, chest pain, dyspnea, etc.) that are caused by locoregional growth of primary tumor [3,4].

Recent publications have reported that radiotherapy of the primary tumor may prolong the survival time of patients with NSCLC involving limited metastatic lesions, and radiation dose to primary thoracic tumor were associated with survival time [5,6]. Higginson et al. [7] conducted a pooled analysis of 189 NSCLC patients with stage IIIB or stage IV, who had never received radiotherapy, from nine prospective clinical studies, and revealed that intrathoracic disease burden had prognostic significance, patients with bulky central disease, bronchial/vascular compression, and/or pulmonary symptoms had worse overall survival after first-line, platinum-based chemotherapy. These results suggest that patients with stage IV disease may benefit from thoracic radiation. Chemotherapy is the main treatment for stage IV NSCLC. However, the research on thoracic three dimensional radiotherapy with chemotherapy for stage IV NSCLC has been limited, and more studies are needed to confirm the outcomes of this treatment modality [8,9]. Therefore, we retrospectively analyze the survival outcomes and prognostic factors in stage IV NSCLC patients who received at least four cycles of chemotherapy and at least 40 Gy of thoracic radiation to primary tumor.

## Methods

### Patient selection and pretreatment evaluation

Ninety-three patients with stage IV NSCLC and fulfilled all of the following criteria have been included in this study. The inclusion criteria were as follows: (1) pathologically or cytologically confirmed diagnosis of NSCLC; (2) newly diagnosed stage IV disease according to the staging system of the 2002 American Joint Committee on Cancer (AJCC); (3) age between 18 and 80 years; (4) Karnofsky Performance Status (KPS) score ≥ 70, as well as a weight loss of no more than 10% during the six months prior to therapy; (5) patients had adequate bone marrow function, liver function and renal function; (6) no radiotherapy or chemotherapy contraindications; (7) the primary thoracic tumor received radiation of at least 40 Gy; (8) thoracic radiation using either three-dimensional conformal radiation therapy (3D-CRT) or intensity-modulated radiation therapy (IMRT); (9) treatment with at least four cycles of systemic chemotherapy; and (10) limited metastatic disease (≤5 sites). The exclusion criteria were as follows: (1) history of thoracic operation, radiotherapy or chemotherapy; (2) pregnancy or lactation; (3) previous malignancy or other concomitant malignant disease. The Institutional Review Board of the Affiliated Hospital of Guiyang Medical College and Guizhou Cancer Hospital China approved this study, and the informed consent was obtained from all patients.

Pretreatment evaluation included a complete physical examination, hematologic and biochemistry profiles. Fiberoptic bronchoscopic examination and contrast-enhanced computed tomography (CT) of chest were performed to accurately evaluate the extent of the primary tumor and regional lymph nodes. Bone scan, contrast-enhanced CT of the abdominal region, and magnetic resonance imaging of head were routinely used to detect distant metastases. Additional investigations were performed if indicated.

### Radiotherapy protocol

All patients were immobilized in the supine position with a T bar, wing board, and Vac-lock cradle. Images with contrast were obtained from the computed tomography simulator for treatment planning purpose. All patients were scanned with serial 5-mm slices from the hyoid bone through the third lumbar vertebra. IMRT or 3D-CRT treatment plans was designed for patients using the ADAC pinnacle^3^ planning system (version 7.4f) and dose distribution was computed with tissue heterogeneity correction. The gross tumor volume (GTV) included thoracic primary tumours and hilar or mediastinal lymph nodes with a short-axis diameter of at least 1 cm on CT, and the planning target volume (PTV) was defined as the GTV plus a 1.5-cm margin for setup uncertainty and respiratory motion. Radiation was delivered with a linear accelerator using 6 MV photons. V20 (percentage of the total lung volume receiving ≥20 Gy), the maximal point dose of spinal cord and mean esophagus dose was required ≤32%, 50 Gy and ≤35 Gy, respectively, for individual treatment plan. Patients received late-course accelerated hyperfractionated radiotherapy (LCAHRT) to thoracic primary site with 3D-CRT or IMRT techniques. The first course of radiation given in 2 Gy per fraction, 5 days a week for a dose of 40 Gy, LCAHRT was delivered in 2 fractions of 1.5 Gy with an interval of 6–8 h per day. A radiation prescription dose of 60–70 Gy were decided to give to patients; if individual tolerability is not acceptable, lower doses of at least 40 Gy should be given. The PTV should be covered by at least the 90% isodose surface. Under the acceptable radiation dose to the normal tissue, radiation dose to thoracic primary tumor can be escalated to 74Gy. Thoracic radiation treatment was implemented concurrently with chemotherapy. All patients received thoracic radiation at least a dose of 40 Gy in 20 fractions (5 days each week). The fractionated radiotherapy dose for the metastatic tumors ranged from 3–10 Gy/fraction with 1 fraction/day, and the total prescribed radiotherapy dose for metastatic lesions ranged from 20–60 Gy. Concurrent thoracic radiation to primary tumor was given within one week following the start of chemotherapy, and radiation to metastatic lesions was implemented concurrently or sequentially with chemotherapy.

### Chemotherapy protocol

Platinum-based doublets chemotherapy was used for all patients, the selection of regimens was according to prior studies [10,11]. The commonly used regimens and usage are as following: 135 mg-175 mg of paclitaxel (P) per square meter of body-surface area (mg/m^2^) or 75 mg/m^2^ of docetaxel (D) on day 1, followed by 80 mg/m^2^ of cisplatinum (C) or carboplatin (Cb) at a dose of 300 mg/m^2^-350 mg/m^2^ were administrated on day 2, and vinorelbine (V) at a dose of 25 mg/m^2^ was administered on days 1 and 8 during thoracic radiotherapy every 21–28 days. After completion of thoracic radiotherapy, patients demonstrating a response or stable disease continued chemotherapy up to four to six cycles, whereas patients who experienced progressive disease or unacceptable toxicity were transferred to second-line therapy. Platinum and taxane-based were main regimens used in current study, PC or PCb regimens were utilized in 38 cases; DC regimens in 51 cases, VC regimen in 4 cases. In total, 89 patients received four cycles of chemotherapy, and 4 patients received five cycles of chemotherapy.

### Statistical analysis

The Statistical Package for Social Sciences, version 13.0 (SPSS, Chicago, IL) was used for statistical analysis. The Kaplan-Meier method was used to calculate the overall survival. The log–rank test was used to compare the survival curves. Multivariate cox regression analyses was used to test independent significant prognostic factors for OS. All statistical tests were two-sided, and p < .05 was considered statistically significant.

## Results

### Survival analysis

Totally, 93 patients were included in this study, the clinical characteristics of the 93 patients are detailed in Table 1. The male/female ratio was nearly 2.6:1.0 and the patient age range were 28 to 78 years (median, 57). The T and N stages were as follows: 32 cases T_1−2_, 61 T_3−4_, and 15 N_0−1_ and 78 N2-3. Fifty-six (60.2%) patients had metastasis in only one site, 37 (39.8%) patients in ≥2 sites. The most common site of metastatic disease at diagnosis was the bone (55.9% of patients), 34 (36.6%) patients had lung metastasis, and 23 (25.8%) patients had metastasis in brain.

**Table 1:** **Clinical characteristics (93 cases)**

**Characteristic**	**No. (%)**
Gender	
Male	67(72.0)
Famale	26(28.0)
Age (years)	
median(range)	57(28–78)
<60	56(60.2)
≥60	37(39.8)
Pathological type	
Squamous carcinoma	27(29.0)
Adenocarcinoma	58(62.4)9
others	8(8.6)
Karnofsky performance status	
≤80	64(68.8)
>80	29(31.2)
T stage	
T_1–2_	32(34.4)
T_3–4_	61(65.6)
N stage	
N_0–1_	15(16.1)
N_2–3_	78(83.9)
Gross tumor volume (cm^3^)	
median(range)	170(35.8-627.6)
<170	45(48.4)
≥170	48(51.6)
Metastasis status	
Multiple organs	37(39.8)
Brain	15(16.1)
subcutaneous nodules	2(2.2)
Distant lymph nodes	5(5.4)
Lung	21(22.6)
Bone	24(25.8)
Liver	5(5.4)
Adrenal	5(5.4)
Single organ	56(60.2)
Brain	9(9.7)
subcutaneous nodules	2(2.2)
Distant lymph nodes	3(3.2)
Lung	13(14.0)
Bone	28(30.1)
Liver	1(1.0)
Radiation dose(Gy) to primary tumor	
median(range)	63(40–74)
<63	29(31.2)
≥63	64(68.8)
Response of primary tumor	
Complete response	5(5.4)
Partial response	66(71.0)
Stable	18(19.4)
Progressive	4(4.3)
No. of chemotherapy cycle	
4	89(95.7)
5	4(4.3)
Radiotherapy to metastases	
Yes	50((53.8)
No	43(46.2)

The follow-up periods ranged from 4.0-80.0 months, with a median follow-up period of 14.0 months. By the last follow-up, 88 patients was dead, whereas five patients remained alive, the survival times of these five patients was 34.0-80.0 months, with a median survival time (MST) of 64.0 months. For all patients, MST was 14.0 months (95% confidence interval (CI), 11.44-16.60), the 1-year, 2-year, and 3-year survival rates were 54.8%, 20.4%, and 12.9%, respectively. Among 61 of the patients who died, the patterns of tumor recurrence and persistence were evaluated: two cases had progressive disease in primary tumor without developing any new metastases, 3 cases had progressive disease in primary tumor with new metastases, 4 cases had progressive disease in primary tumor and initially metastatic lesions, 48 cases had new metastases in initially involved or uninvolved organs, and 4 cases had no tumor progression (including three patients who died of lung infections and one patient who died of gastrointestinal bleeding). In this study, only 3 patients received treatment of epidermal growth factor receptor (EGFR) tyrosine kinase inhibitors (TKI); the overall survival time of these 3 patients was 26.0, 27.0, and 44.0 months, respectively.

### Prognostic factors analysis

**Figure 1:**
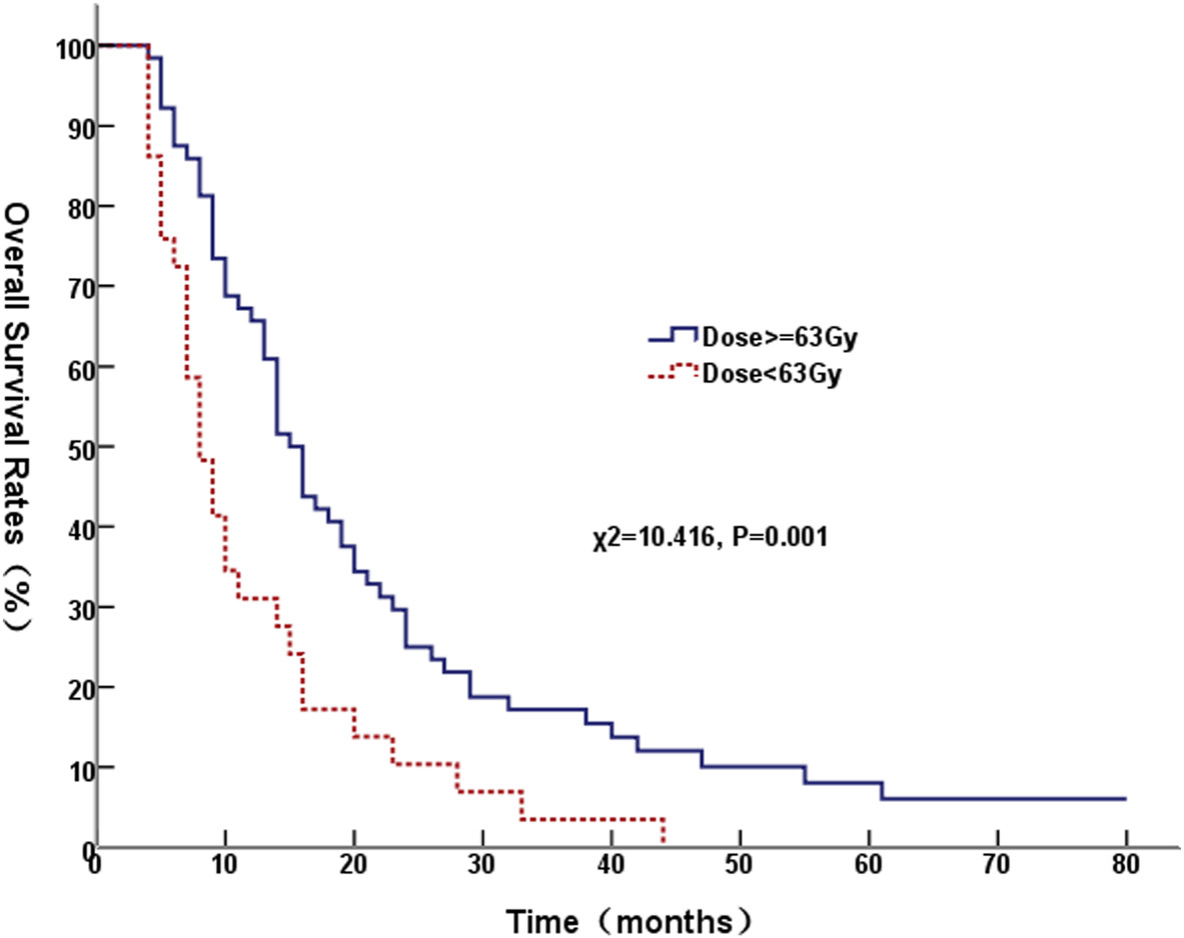
**Comparison of overall survival curves at different radiation dose.**

**Figure 2:**
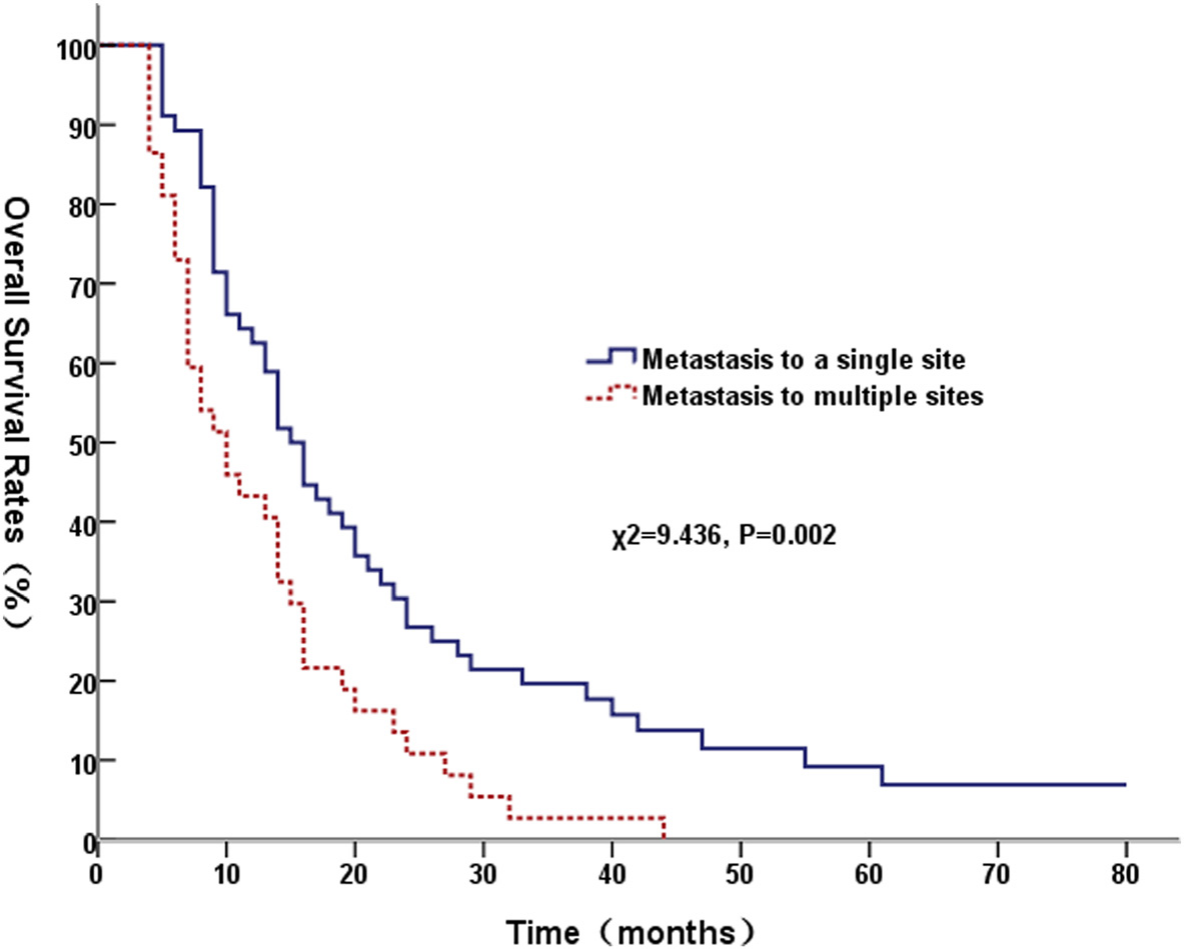
**Comparison of overall survival curves between a single site metastases and multi-site metastases.**

Univariate analysis showed that receipt of at least 63 Gy to the primary tumor (Figure 1), metastasis to a single site (Figure 2), GTV <170 cm^3^, and had response of primary tumor were significantly associated with better OS; and KPS score > 80 was marginally with better OS (*P* = 0.055). Table 2 showed univariate analysis of various potential prognostic factors on predicting OS.

**Table 2:** **Univariate analysis for overall survival**

**Varable**	**No.**	**1-year**	**2-year**	**3-year**	**P value**
Gender					
Male	67	53.7	25.4	11.9	0.833
Female	26	57.7	19.2	15.4	
Age (years)					
<60	56	51.8	16.1	8.9	0.152
≥60	37	59.5	27.0	18.9	
KPS					
≤80	64	50.0	17.2	7.8	0.055
>80	29	58.6	27.6	24.1	
Pathological type					
Non-Squamous	27	55.9	17.7	7.7	0.160
Squamous	66	47.3	10.9	10.9	
GTV (cm^3^)					
<170	45	66.7	31.1	20.0	0.020
≥170	48	43.8	10.4	6.2	
T stage					
T1-2	32	59.4	25.0	18.8	0.339
T3-4	61	52.5	18.0	9.8	
N stage					
N0-1	15	66.7	33.3	26.7	0.229
N2-3	78	52.6	19.2	10.3	
Metastatic status					
Single site	56	62.5	26.8	19.6	0.002
Multiple sites	37	43.2	10.8	2.7	
Thoracic radiation dose					
<63Gy	29	31.0	10.3	3.4	0.001
≥63Gy	64	65.6	25.0	17.2	
Response of primary tumor					
CR	5	80.0	40.0	20.0	0.000
PR	66	59.1	21.2	12.1	
NC	18	44.4	16.7	16.7	
PD	4	0	0	0	
Radiation to metastases					
Yes	50	58.9	22.0	14.0	0.759
No	43	51.2	18.6	11.6	

According to GTV size, for patients with tumor volume ≥170 cm^3^, the MST was 14.0 months (95% CI, 12.7–15.3) for those received radiation ≥63 Gy, and the MST was 8.0 months (95% CI, 5.9-10.1) for those received radiation <63Gy (χ^2^ = 5.609, P = 0.018); for patients with tumor volume < 170 cm^3^, radiation dose ≥ 63 Gy had a trend towards a better OS (χ^2^ = 2.376, P = 0.123). We then subdivided the whole group into patients with metastasis at a single site and multiple sites. For patients with metastasis to a single sites, the MST was 16.0 months (95% CI, 12.45-19.55) for patients who received radiation dose ≥63 Gy, and 10.0 months (95% CI, 8.33-11.67) for patients who received radiation dose < 63 Gy (χ^2^ = 4.733, P = 0.030). For patients with metastasis to multiple sites, patients who received ≥ 63 Gy had a MST of 14.0 months (95% CI, 9.99-18.01), whereas the patients who received <63 Gy had a MST of 7.0 months (95% CI, 6.01-7.99) (χ^2^ = 3.488, P = 0.062). Patients with and without brain metastasis had similar OS (χ^2^ = 2.502, P = 0.114).

The multivariate analysis showed that the number of metastatic sites, and radiation dose of the primary thoracic tumor were independent prognostic factors for OS, and the volume of primary tumor was marginally correlated with OS (Table 3).

**Table 3:** **Multivariate analysis for overall survival**

**Variable**	**HR**	**95.0% confidence interval**		***P*** **value**
		**Lower**	**Upper**	
Sex (female vs. male)	1.265	0.701	2.284	0.435
Age (<60 y vs. ≥ 60y)	0.953	0.552	1.646	0.864
Karnofsky performance status (≤80 vs. >80)	0.779	0.425	1.428	0.420
Pathological type (Squamous vs.Non-Squamous )	1.314	0.785	2.199	0.298
Tumor volume (per cm^3^ incerase)	1.002	1.000	1.003	0.054
T stage (T3-4 vs.T1-2)	1.209	0.677	2.157	0.521
N stage (N2-3 vs.N0-1)	1.015	0.435	2.366	0.973
Metastasis status (multi- vs. single)	1.765	1.032	3.018	0.038
Thoracic radiation dose (≥63 Gy vs. <63 Gy)	0.547	0.333	0.897	0.017
Response of primary tumor (SD + PD vs. CR + PR)	1.232	0.574	2.642	0.592
Radiotherapy to metastases (yes vs. no)	0.890	0.511	1.551	0.681

## Discussion

The response rate for platinum-based doublet chemotherapy for stage IV NSCLC is approximately 30%-40%, and this treatment produces a MST of 8.0-10.0 months. Different third-generation chemotherapy regimens have similar efficacies, indicating that the efficacy of a chemotherapeutic approach has reached a plateau [11,12]. This study sought to investigate whether combining systemic chemotherapy with radiotherapy of the primary thoracic tumor could further improve survival. Compared with historical data [11,12], the survival time of patients treated with this combined therapy in current study were increased. The multivariate analysis demonstrated that radiation ≥63 Gy for the primary thoracic tumor and metastasis to only a single site are independent prognostic factors for better survival.

Systemic chemotherapy is the primary treatment for stage IV NSCLC, and four to six cycles of chemotherapy is recommended [2]. Radiotherapy can effectively ameliorate the various symptoms caused by the primary tumor, including hemoptysis, pain, coughing, and dyspnea; however, patients often only receive palliative dose of radiotherapy [9,13,14]. The current study revealed that the MST of the patients who received a definitive radiation ≥63 Gy to the primary thoracic tumor was 15.0 months, which is significantly higher than the MST of 8.0 months among the patients received a dose <63 Gy. A multivariate analysis indicated that a dose ≥ 63 Gy is an independent prognostic factor for survival, demonstrating that treatment with a radical dose of radiotherapy to the primary tumor can prolong the survival time of patients with stage IV NSCLC. Nearly 50% of stage IV NSCLC patients develop local recurrence in initially involved sites, local control and status of primary tumor was associated with OS [7,15,16]. These findings suggest that local control of the primary tumor plays an important role in prolonging the survival of patients with stage IV NSCLC.

Consistent with Lopez et al. [6], our result also showed that patients with a lower tumor volume had better outcomes. In subgroup analysis, among patients with larger tumors (GTV > 170 cm^3^) or metastasis to only a single site, we found that patients who received ≥63 Gy had a significantly improved OS than those who received < 63 Gy. Our findings suggest that primary tumor volume and metastasis status may be used as a criterion for selecting patients for aggressive radiation therapy in this setting.

In the current study, an analysis of the outcomes for 61 patients with confirmed causes of death revealed that 57 patients died of tumor progression, 48 patients died of new metastases, 7 patients died due to local recurrences accompanied by distant metastases, and only 2 patients died of local recurrences alone. These results demonstrate that aggressive radiotherapy can reduce the risk of death from local recurrence. Moreover, the investigation of more effective systemic treatment regimens is important for improving the prognosis of stage IV NSCLC. Lopez et al. [6] found that the radiation dose of the primary thoracic tumor was associated with survival among patients with stage IV NSCLC, suggesting that the administration of aggressive radiotherapy to the primary tumor can improve survival. Arrieta et al. [5] reported that in NSCLC patients with metastases only in the brain, if the absence of progression was assessed after receiving two cycles of systemic chemotherapy and received radiation of the metastatic lesions in the brain, the survival outcomes could be improved by the administration of active concurrent chemoradiotherapy of the patients’ thoracic primary tumors; in particular, this therapeutic approach achieved an MST of 31.8 ± 15.8 months.

Hellman et al. [17] proposed a notion is that of oligometastases in 1995, oligometastases is the state in which the patient shows distant relapse in only a limited number of regions. In the current study, a multivariate analysis indicated that metastasis to only a single site was an independent prognostic factor for survival. The subgroup analysis demonstrated that radiotherapy with a high dose radiation (≥63 Gy) to primary thoracic tumor may not only provide survival benefits to patients with metastasis to a single site but also have a trend to improve the survival for patients with metastases in multiple sites. The current study data suggest that NSCLC patients with limited metastatic disease could derive a greater benefit from radiotherapy for their primary tumors. Several retrospective studies have also showed that patients with imited stage IV NSCLC can benefit from aggressive radiotherapy of primary tumors [5,6,18]. Mehta et al. [16] reported that 74% of stage IV NSCLC patients had metastases confined to one or two sites and 50% had ≤3 metastatic sites at initial diagnosis, and 50% had stable or progressive disease in initially involved sites without developing any new metastatic tumors at last follow-up. These data suggest that a subset of patients who present with metastatic NSCLC may not have widely disseminated disease, and local treatment combined with systemic therapy might be beneficial in these patients.

## Conclusions

In summary, newly diagnosed NSCLC patients with metastasis may benefit from aggressive radiotherapy (of ≥63 Gy) to primary thoracic tumor based on systemic chemotherapy. Patients with metastasis to only a single site and smaller tumor volume tend to benefit most from this aggressive treatment modality, although this approach also have a trend to provide survival benefits for patients with metastases in multiple sites. The administration of aggressive radiotherapy to control primary tumors may play an important role in improving survival of NSCLC patients with metastases. Because of the retrospective nature of current study, a randomized trial is necessary to evaluate the causal effect of radiation dose on OS.
